# Quality of Life and Social Functioning during Treatment of Recent Hepatitis C Infection: A Multi-Centre Prospective Cohort

**DOI:** 10.1371/journal.pone.0150655

**Published:** 2016-06-29

**Authors:** Joseph S. Doyle, Jason Grebely, Tim Spelman, Maryam Alavi, Gail V. Matthews, Alexander J. Thompson, Gregory J. Dore, Margaret E. Hellard, ATAHC Study Group

**Affiliations:** 1 Centre for Population Health, Burnet Institute, Melbourne, Australia; 2 Department of Infectious Diseases, Alfred Hospital, Melbourne, Australia; 3 School of Population Health and Preventive Medicine, Monash University, Melbourne, Australia; 4 Viral Hepatitis Clinical Research Program, Kirby Institute, UNSW Australia, Sydney, Australia; 5 Infectious Diseases Unit, St Vincent’s Hospital, Sydney, Australia; 6 Department of Gastroenterology, St Vincent’s Hospital Melbourne, Melbourne, Australia; 7 Department of Medicine, University of Melbourne, Melbourne, Australia; Kaohsiung Chang Gung Memorial Hospital, TAIWAN

## Abstract

**Aim:**

Despite effective treatment for recent hepatitis C (HCV) infection, side-effects and adherence concerns limit its use among people who inject drugs (PWID). This study evaluated health-related quality of life (HRQoL) and social functioning following infection and during recent HCV treatment.

**Methods:**

The Australian Trial of Acute Hepatitis C studied the natural history and treatment of recent HCV infection. HRQoL (SF-12v2) and social functioning (Opiate Treatment Index score) were measured over 48 weeks and their impact on treatment uptake, adherence and virological response were assessed.

**Results:**

Of 163 participants, 111 received treatment (HCV n = 74, SVR 55%; HCV/HIV n = 37, SVR 74%). 116 (71%) were male, 124 (76%) ever injected drugs, with 55 (36%) injecting recently and 28/55 (51%) reported needle/syringe sharing. At baseline, median physical and mental HRQoL was 54 units (IQR 46–58) and 46 (35–54) (reference median: 50), respectively, and median social functioning score was 11 units (7–17). Higher social function (<10 vs ≥15) predicted increased treatment uptake (AOR 3.43, 95%CI 1.01–11.6, p = 0.048) and higher SVR (AOR 5.11, 95%CI 1.30–20.15, p = 0.020). After adjustment, treated participants had lower physical (-4.90 units, 95%CI -6.33 to -3.48, p<0.001) and mental HRQoL (-3.7 units, 95%CI -5.55 to -1.86, p<0.001) at on-treatment visits, but HRQoL returned to baseline levels during follow-up.

**Conclusions:**

Social functioning can predict recent HCV treatment uptake and SVR. Efforts to maximise social stability may improve treatment response. Pegylated-interferon treatment is associated with reduced HRQoL on-treatment in an already vulnerable population of PWID that would be better served by interferon-free regimens particularly in treated target at PWID to prevent transmission.

**Trial Registration:**

ClinicalTrials.gov NCT00192569

## Introduction

Treatment of recently acquired hepatitis C virus (HCV) infection using pegylated-interferon (PEG-IFN) is effective and results in a sustained cure for most patients [1–6]. However, PEG-IFN therapy is also characterised by considerable toxicity, including neuropsychiatric side-effects, lethargy, influenza-like symptoms, and cytopenias, all of which necessitate individualised decisions to initiate treatment [7–10]. The potential to adversely affect quality of life, social stability and other co-morbidities creates complexity and limits use of PEG-IFN. While interferon (IFN)-free therapies promise better and simpler treatment, access remains restricted largely due to cost and licensing limitations. Sustained, large scale treatment of recent HCV infection among people who inject drugs (PWID) could have important prevention benefits [11].

Chronic HCV infection is known to adversely influence health-related quality of life (HRQoL) [12–18]. HRQoL changes in HCV may be related to cirrhosis [14–17], since the natural history of HCV infection is to remain largely asymptomatic, except among a minority who display acute seroconversion illness [19]. Chronic HCV treatment with PEG-IFN has also been shown to impair HRQoL [12, 20, 21], and conversely sustained virological response (SVR) may improve HRQoL [14, 21, 22]. However, HRQoL following recent HCV infection and during early treatment remains poorly understood. Successful early treatment has the potential to avoid future declines in health status as well as prevent transmission.

Social stability measures have been used among populations of people who inject drugs (PWID) and applied previously to HCV [23, 24]. Social functioning relates to stable accommodation, employment and inter-personal relationships. These factors may influence treatment deferral decisions and manifestation of side-effects during HCV treatment [25–27]. Given the burden of HCV-related morbidity and mortality globally, a better understanding of non-virological outcomes following recent HCV treatment is important. Social stability may also reflect willingness to engage in care and treatment, which will remain an ongoing challenge in recent HCV infection when newer direct-acting antivirals are available. Quality of life and social functioning data may aid guideline development and public health decision-makers to appraise and cost future treatment strategies for recent HCV. They will also assist in directing social supports needed to maximise treatment uptake, retention in care, and future treatment as prevention strategies.

The Australian Trial in Acute Hepatitis C (ATAHC) was a prospective, non-randomised trial of the natural history and treatment of recently acquired HCV infection, and predominantly recruited PWID [5]. This cohort provided an opportunity to examine how HRQoL and social functioning varies particularly among PWID, who are often excluded from other clinical trials. The aims of this study were to evaluate HRQoL and social functioning following recent HCV infection and during treatment. Further, it aimed to explore the impact of HRQoL and social functioning on treatment uptake, adherence and SVR. It was hypothesised that HRQoL and social functioning are adversely affected during PEG-IFN-based therapy, and that participants with higher HRQoL and social functioning have higher treatment uptake, adherence and SVR.

## Methods

### Study design

The ATAHC Study cohort recruited 163 individuals with recently acquired HCV infection between 2004 and 2008 from an Australian network of tertiary hospitals (n = 13) and general practice (n = 3), as previously reported [5]. Documented HCV infection was based on either of the following criteria:

1. Acute clinical HCV infection, defined as symptomatic seroconversion illness or medically documented jaundice or alanine aminotransferase (ALT) level greater than ten times the upper limit of normal (>400 IU/L) with exclusion of other causes of acute hepatitis, and HCV RNA detection or high-risk exposure within the preceding four months; or
2. Asymptomatic HCV infection with seroconversion, defined by a negative anti-HCV test in the two years prior to the initial positive anti-HCV antibody or HCV RNA test.

All participants with detectable HCV RNA during the first 12 weeks of enrolment were eligible for treatment, with no exclusions for active injecting drug use or alcohol intake. All participants were followed for 12 weeks to observe for spontaneous clearance before treatment initiation. All ATAHC participants are included in this analysis of HRQoL and social functioning.

### Ethics statement

Participants provided written informed consent. The study was approved by St Vincent’s Hospital Sydney Human Research Ethics Committee. The study was registered at clinicaltrials.gov (NCT00192569).

### HCV treatment

Participants were treated using PEG-IFN alpha-2a (180μg weekly) in HCV mono-infection, or in combination with ribavirin (800-1200mg daily) in HIV co-infection, for 24 weeks duration.

### Study assessments

Questionnaires were administered at enrolment and every 12 weeks among all participants to obtain clinical information and measurement of behaviour (*ever* injected, *current injecting* in last six months, *recent injecting* in last month), social functioning (Opiate Treatment Index Social Functioning Scale [23]), health-related quality of life (SF-12v2, Quality Metric, Lincoln RI, USA) and psychological parameters (Mini-International Neuropsychiatric Interview [28] and the Depression Anxiety Stress Scale [29]). In addition, treated participants had clinical visits every two weeks from treatment initiation to week 8, then four weekly until end of treatment. All participants were followed for a maximum of 144 weeks. Data up to 48 weeks (ie SVR visit) were included in this analysis. HCV RNA assessment was performed using combined qualitative/quantitative assays (COBAS AmpliPrep/COBAS Taqman, Roche, USA, lower limit of detection 15 IU/ml). HCV genotype was determined by line-probe assay (Versant LiPa1/LiPa2; Bayer, Australia). *IFNL4* (also known as *IL28B*) genotype was determined by sequencing of the rs12979860 single-nucleotide polymorphism [30].

### Study definitions

*Health-related quality of life* measurement used the SF-12v2 Health Survey, which is a validated instrument for measuring HRQoL generating a physical component summary (PCS) and mental component summary (MCS)[31], is interchangeable with the SF-36 Health Survey [32], and has been applied in HCV previously [12, 33]. PCS and MCS scores range from 0–100, and are standardised using US population norms (updated 2009) to a population mean of 50 and standard deviation of 10. *Higher* scores indicate *better* HRQoL. *Social functioning* was calculated from the Social Functioning Scale of the Opiate Treatment Index (OTI), which addresses employment, accommodation stability, social support and inter-personal relationships [23]. It has been validated among opiate using populations in Australia [23]. Scores range from 0–48 with *higher* OTI scores indicating *lower* social stability.

*Recently acquired HCV infection* was defined as either acute (<6 months) or early chronic (6–24 months). *Treatment uptake* was defined as receiving at least one dose of PEG-IFN among all participants with HCV RNA detectable during the screening period (n = 145). *Adherence* was defined as receiving at least 80% of scheduled PEG-IFN doses for at least 80% of scheduled treatment period. For patients who terminated treatment at 12 weeks due to non-virological response, the scheduled treatment duration was 12 weeks. *SVR* was defined as undetectable HCV RNA at 24 weeks post-treatment; the proportion achieving SVR was calculated based on all participants who received at least one dose of PEG-IFN.

### Study Outcomes

The primary outcomes of the study were: (1) HRQoL and social functioning scores at baseline; and (2) longitudinal HRQoL and social functioning scores across study visits. Secondary outcomes were treatment uptake, adherence and SVR. Where HRQoL was explored as a binary variable, it was categorised as high or low based on the population median score of 50 units. Where social functioning was explored as a categorical variable, it was divided into tertials based approximately on data spread in the cohort since there are no commonly accepted cut-off points. Key hypotheses under investigation were that HCV spontaneous clearance and HIV co-infection would be associated with higher HRQoL and social functioning.

### Statistical analysis

Categorical characteristics of participants at baseline were summarised using frequency and percentage and analysed by treatment outcome using the χ^2^ test. Baseline differences in HRQoL and social functioning were described using median and interquartile range, and compared using the Wilcoxon rank-sum test. Predictors of HRQoL and social functioning at baseline were analysed using logistic regression to determine crude and adjusted odds ratios (OR) with 95% confidence intervals. Potential covariates for exploration (in all models) were identified *a priori* including age, gender, education, employment, accommodation, opiate substitution therapy, injecting behaviour (past, current and recent injecting, sharing needles and equipment), alcohol use (at all in the past month; >2 drinks/day), major depression, duration of infection, clinical presentation (symptomatic versus asymptomatic), and *IFNL4* genotype. Multivariable model development included covariates significant at the 0.20 level, and key hypotheses under investigation. Covariates that were not independent of outcome under exploration were excluded eg depression with mental HRQoL; accommodation and employment with social functioning. A backwards stepwise approach sequentially eliminated variables subject to the result of a likelihood ratio test, and the final model was evaluated using a Hosmer-Lemeshow goodness-of-fit test.

Longitudinal HRQoL and social functioning scores were described at baseline (week 0), on-treatment (weeks 12–24), and post-treatment (weeks 36–48) time points. Changes from baseline to on-treatment and post-treatment were compared using the rank-sum test, and across all treatment stages using the Kruskal-Wallis test. Factors associated with longitudinal HRQoL and social functioning score at each study visit were investigated using generalised estimating equations. Selection of the model correlation structure and model selection was undertaken using the Quasi-likelihood Information Criterion. Treatment was included as a binary variable (treated vs untreated) in the final adjusted model, but a sensitivity analysis examined treatment effects at three time points–on-treatment vs baseline, and post-treatment vs baseline–adjusting for the same independent predictors.

The effect of HRQoL and social functioning on treatment uptake (among those RNA detectable at enrolment therefore eligible for treatment), adherence and SVR (among those treated) was explored by logistic regression. Using bivariate data previously reported from the ATAHC cohort, each final model was adjusted for factors known to be independently associated with outcome. Treatment uptake was adjusted for duration of infection and HCV RNA log viral load at screening [34]. Adherence was adjusted for education level [35]. SVR was adjusted for OST use, gender, *IFNL4* genotype, HCV RNA level, and duration of infection [5]. Differences were considered significant at the 0.05 level using two-sided p values. All analyses were conducted using Stata (v13.1, Stata Corporation, College Station, TX, United States).

## Results

### Participant characteristics

Overall, 163 participants were enrolled between June 2004 and February 2008. The majority were male (116, 71%) with mean age 34 years (Table 1) and 50 (31%) were HIV co-infected. At baseline, 124 (76%) reported ever injected drugs, 55 (36%) reported injecting within the last month and 28/55 (51%) reported needle/syringe sharing One hundred and eleven participants received treatment, 18 spontaneously cleared prior to enrolment, 12 spontaneously cleared after enrolment, and 22 remained untreated and viraemic. Compared with untreated participants, those commencing treatment were more likely to have stable full/part-time employment (21% vs 47%), post-secondary education (29% vs 46%), consume alcohol in the last month (42% vs 66%), and less likely to inject drugs recently (46% vs 28%) or have depression (29% vs 9%). Overall treatment outcomes have been published previously [5, 36]. In brief, among HCV monoinfected participants (n = 74), 55% achieved SVR while among HCV/HIV co-infected (n = 37), SVR was 74%. Median and maximum duration of follow up were 48 and 144 weeks, respectively.

**Table 1:** Characteristics of participants in Australian Trial in Acute Hepatitis C, by treatment group and HCV RNA status at baseline.

Characteristic	Total population	Treated	Untreated	
			RNA positive at enrolment	RNA negative at enrolment
**Total**, n	163	111	34	18
**Age**, years, mean (sd)	34.1(±9.6)	34.3(±10.4)	34.6 (±9.0)	32.1 (±8.3)
**Male**, n (%)	116 (71%)	83 (75%)	22 (65%)	11 (61%)
**Body mass index,** kg/m^2^, median (IQR)	23.3 (21–26)	23.3 (21–25)	22.4 (20–26)	25.6 (23–31)
**Caucasian ethnicity**, n (%)	149 (91%)	99 (89%)	33 (97%)	17 (94%)
**Tertiary education or greater**, n (%)	66 (40%)	51 (46%)	9 (26%)	6 (33%)
**Regular employment**, n (%)	63 (39%)	52 (47%)	9 (26%)	2 (11%)
**Accommodation stable**, n (%)	140 (86%)	97 (87%)	29 (85%)	14 (78%)
**Opiate substitution therapy, current**, n (%)	22 (14%)	12 (11%)	6 (18%)	4 (22%)
**Mode of HCV transmission**, n (%)				
** **Injecting behaviour	119 (73%)	77 (69%)	26 (76%)	16 (90%)
** **Sexual behaviour	29 (18%)	25 (23%)	3 (9%)	1 (5%)
** **Other or unknown	15 (9%)	9 (8%)	5 (15%)	1 (5%)
**Injecting drug use**, n (%)				
** **Ever	124 (76%)	84 (76%)	28 (82%)	12 (67%)
** **Within last 6 months	102 (63%)	69 (62%)	23 (68%)	10 (57%)
** **Within last 1 month	55 (36%)	31 (28%)	16 (47%)	8 (44%)
** **Borrowed syringe or needle/last month	9/55 (16%)	7/31 (24%)	2/16 (13%)	0/8
** **Shared injecting equipment/last month	28/55 (51%)	15/31(48%)	10/16 (53%)	3/8 (38%)
**Alcohol use**, n (%)				
** **Any drinks in last month (n = 150)	95 (58%)	73 (66%)	14 (41%)	8 (44%)
** **>2 drinks/day in last month, (n = 148)	18 (11%)	10 (9%)	3 (9%)	5 (28%)
**Quality of life** (n = 151)				
** **Physical component score, median (IQR)	53.6 (46–58)	54.3 (46–58)	52.1 (46–57)	50.4 (42–59)
** **Mental component score, median (IQR)	45.8 (35–54)	47.8 (38–55)	39.2 (32–48)	40.4 (27–53)
**Social functioning score**^**1**^, median (IQR) (n = 146)	13 (8–19)	11 (7–17)	16 (10–20)	17.5 (13–20)
**Major depression current**, n (%)	25 (15%)	10 (9%)	9 (25%)	6 (33%)
**HIV infection**, n (%)	50 (31%)	37 (33%)	11 (32%)	2 (11%)
**Estimated duration of infection, enrolment** weeks, median (IQR)	24 (16–37)	25 (16–42)	19 (11–28)	26 (20–31)
**Presentation of recent HCV**, n (%)				
** **Symptomatic illness	67 (41%)	46 (41%)	12 (35%)	9 (50%)
** **Asymptomatic seroconversion	64 (39%)	41 (37%)	16 (47%)	7 (39%)
** **Acute clinical ALT ≥400 IU/ml	32 (20%)	24 (22%)	6 (18%)	2 (11%)
**HCV RNA, enrolment**, log_10_ IU/mL, median (IQR)	4.5 (3.0–5.8)	5.1 (3.9–5.9)	3.3 (2.8–5.0)	negative
**HCV genotype of primary infection**, n (%)				
** **Genotype 1	75 (46%)	62 (56%)	13 (38%)	-
** **Genotype 2	6 (4%)	4 (4%)	2 (6%)	-
** **Genotype 3	56 (34%)	40 (36%)	16 (47%)	-
** **Genotype 4	1 (1%)	0	1 (3%)	-
** **Missing/not possible	25 (15%)	5 (4%)	2 (6%)	18 (100%)
***IFNL4* genotype**, n (%)				
** **CC rs12979860 allele	78 (48%)	51 (46%)	16 (47%)	11 (61%)
** **Missing	12 (7%)	9 (8%)	2 (6%)	1 (6%)

^1^Higher score indicates lower social functioning and higher social instability

sd, standard deviation; IQR, interquartile range; ALT, alanine transaminase

### HRQoL and social functioning at baseline

HRQoL surveys were completed and scored among 151 participants. At enrolment, median physical and mental HRQoL was 54 units (IQR 46–58) and 46 units (35–54) (population reference: median 50, standard deviation 10 units), respectively. Physical HRQoL was similar between treated and untreated participants, while mental HRQoL was higher among those receiving treatment (48 vs 40, p = 0.006).

In unadjusted analyses, stable full/part-time employment, tertiary education, alcohol consumption in last month, and asymptomatic seroconversion were associated with higher physical HRQoL (S1 Table). In adjusted analyses, employment remained associated with higher physical HRQoL (AOR 4.51, 95% CI 1.90–10.7, p<0.001) and symptomatic acute infection was associated with lower physical HRQoL (AOR 0.25, 95% CI 0.11–0.57, p<0.001) at baseline. Mental HRQoL was associated with stable employment, higher social functioning, and injecting drugs in unadjusted analyses. After adjustment, ever injecting drugs was independently associated with lower mental HRQoL (AOR 0.30, 95% CI 0.13–0.71, p = 0.006).

Social functioning data was complete among 146 participants at enrolment. Median social functioning score was higher among treated than untreated participants (11 units, IQR 7–17 vs 16, IQR 10–20; p = 0.006). In unadjusted analysis, female gender, injecting drugs, needle sharing, and failure to spontaneously clear HCV RNA were associated with lower social functioning (S2 Table), while HIV coinfection and high HRQoL were associated with higher social functioning. After adjustment, ever injecting (AOR 0.18, 95% CI 0.06–0.59, p = 0.004) and sharing needles/equipment (AOR 0.16, 95% CI 0.04–0.74, p = 0.019) remained associated with lower social functioning; HIV co-infection remained associated with higher social functioning (AOR 2.40, 95% CI 1.02–5.66, p = 0.046).

### Treatment uptake

**Fig 1:**
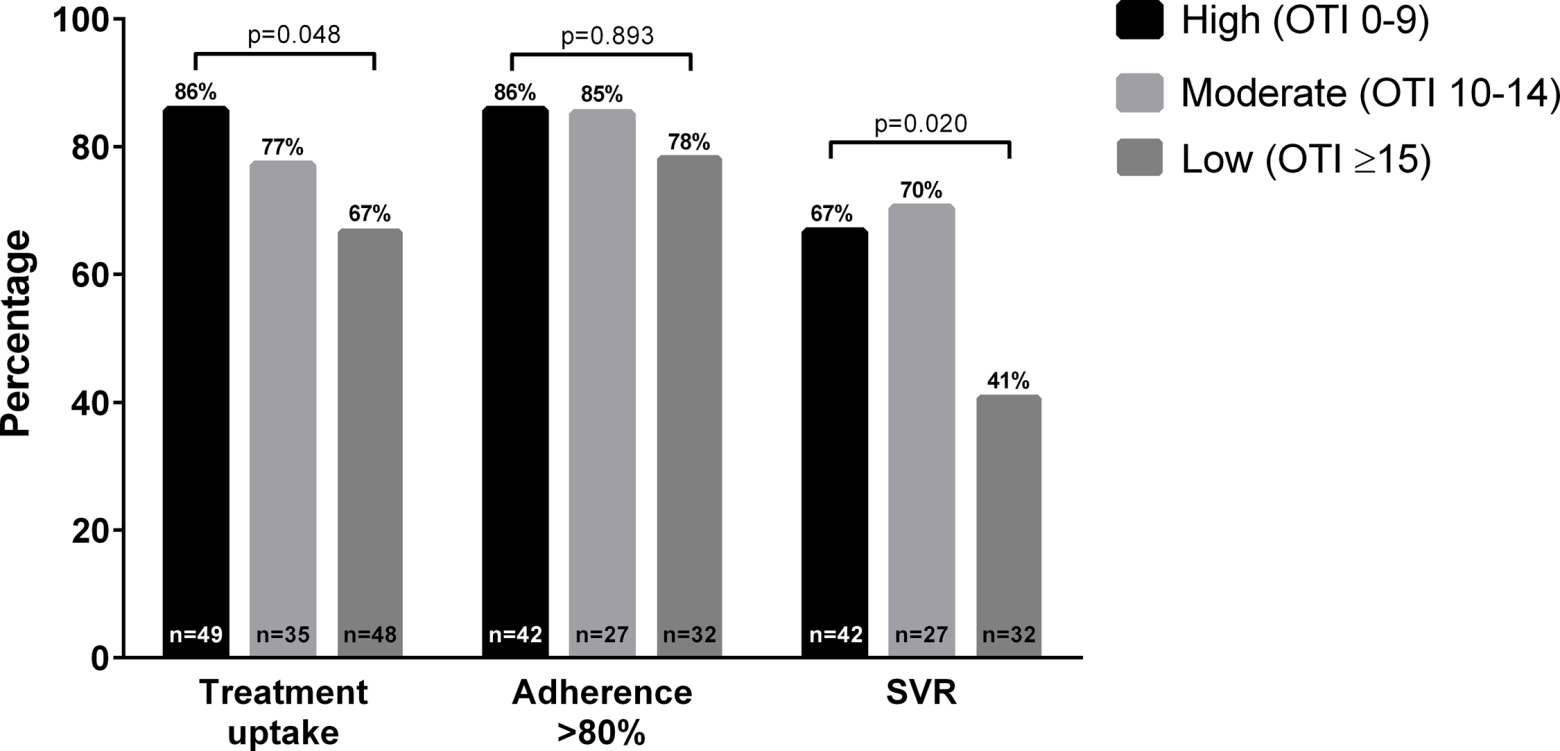
Recent HCV treatment uptake, adherence and sustained virological response by social functioning score. (Opiate Treatment Index score 0–9, 10–14, ≥15) at baseline.

Physical or mental HRQoL did not impact on treatment uptake (S1 Fig). Of those HCV RNA detectable at enrolment (n = 145), treatment uptake was 78% among those with high physical HRQoL (>50^th^ centile), compared to 75% with low physical HRQoL (p = 0.676). After adjustment for HIV, duration of infection and HCV RNA level [34], there was no association between baseline physical HRQoL and treatment uptake (Table 2). Treatment uptake was 88% among those with high mental HRQoL, compared to 71% with low mental HRQoL (p = 0.037). There was a trend toward higher mental HRQoL predicting treatment uptake (AOR 2.60, 95% CI 0.93–7.29, p = 0.069). Social functioning comparing highest to lowest stratum (OTI score <10 vs ≥15), was independently associated with treatment uptake (86% vs 67%; AOR 3.43, 95% CI 1.01–11.64, p = 0.048) (Fig 1).

**Table 2:** Impact of health-related quality of life and social functioning on treatment uptake, adherence and SVR.

Outcome	Characteristic	n (%)	Univariable model		Multivariable model	
			OR (95% CI)	p value	AOR (95% CI)	p value
**Treatment uptake**, n = 145	**Physical HRQoL**					
	Lower (≤50^th^ centile)	19 (75%)	1	–	1	–
	Higher (>50^th^ centile)	68 (78%)	1.19 (0.52–2.73)	0.676	0.86 (0.35–2.13)	0.752^1^
	**Mental HRQoL**					
	Lower (≤50^th^ centile)	62 (71%)	1	–	1	–
	Higher (>50^th^ centile)	42 (88%)	2.82 (1.07–7.47)	0.037	2.60 (0.93–7.29)	0.069^1^
	**Social functioning, binary**					
	Lower (≥12 OTI score)	45 (69%)	1	–	1	–
	Higher (<12 OTI score)	56 (84%)	2.26 (0.98–5.21)	0.055	2.31 (0.91–5.81)	0.077^1^
	**Social functioning, highest v lowest tertile**					
	Lowest (≥15 OTI score)	32 (67%)	1	–	1	–
	Highest (<10 OTI score)	42 (86%)	3.00 (1.10–8.15)	0.031	3.43 (1.01–11.64)	0.048^1^
**Treatment adherence**, n = 111	**Physical HRQoL**					
	Lower (≤50^th^ centile)	27 (75%)	1	–	1	–
	Higher (>50^th^ centile)	59 (87%)	2.19 (0.78–6.12)	0.137	1.60 (0.54–4.77)	0.403^2^
	**Mental HRQoL**					
	Lower (≤50^th^ centile)	54 (87%)	1	–	1	–
	Higher (>50^th^ centile)	32 (76%)	0.47 (0.17–1.32)	0.154	0.43 (0.15–1.26)	0.124^2^
	**Social functioning, binary**					
	Lower (≥12 OTI score)	36 (80%)	1	–	1	–
	Higher (<12 OTI score)	48 (86%)	1.50 (0.53–4.27)	0.447	1.11 (0.37–3.35)	0.851^2^
	**Social functioning, highest v lowest tertile**					
	Lowest (≥15 OTI score)	25 (78%)	1	–	1	–
	Highest (<10 OTI score)	36 (86%)	1.68 (0.50–5.60)	0.398	1.09 (0.29–4.08)	0.893^2^
**Sustained virological response**, n = 111	**Physical HRQoL**					
	Lower (≤50^th^ centile)	23 (64%)	1		1	
	Higher (>50^th^ centile)	40 (59%)	0.81 (0.35–1.86)	0.615	0.88 (0.37–2.10)	0.778^3^
	**Mental HRQoL**					
	Lower (≤50^th^ centile)	41 (66%)	1		1	
	Higher (>50^th^ centile)	22 (52%)	0.56 (0.25–1.26)	0.161	0.62 (0.27–1.44)	0.269^3^
	**Social functioning, binary**					
	Lower (≥12 OTI score)	20 (44%)	1		1	
	Higher (<12 OTI score)	40 (71%)	3.13 (1.37–7.14)	0.007	4.37 (1.52–12.55)	0.006^3^
	**Social functioning, highest v lowest tertile**					
	Lowest (≥15 OTI score)	13 (41%)	1		1	
	Highest (<10 OTI score)	28 (67%)	2.92 (1.13–7.59)	0.027	5.11 (1.30–20.15)	0.020^3^

^1^Adjusted for HIV status and independent predictors of treatment uptake: duration of infection at screening, and HCV RNA log viral load [34].

^2^Adjusted for HIV status and independent predictors of treatment adherence >80%: education level [35].

^3^Adjusted for HIV status and independent predictors of SVR: *IFNL4* genotype, gender, duration of infection, HCV RNA level and current opiate substitution therapy [5].

### Adherence

Adherence to >80% PEG-IFN doses and >80% duration was achieved by 91/111 (82%) participants. Adherence did not differ between those who had higher versus lower physical HRQoL (87% vs 75%) or mental HRQoL (76% vs 87%). Adherence was 86% among those with highest social functioning compared to 78% with lowest social functioning score. After adjustment for HIV and education level [35], neither HRQoL nor social functioning scores were associated with adherence (Table 2).

### Sustained virological response

Of the 111 participants treated, overall SVR was achieved by 60%. There was no significant difference in SVR by higher vs lower physical HRQoL score (59% vs 64%) or mental HRQoL score (52% vs 66%). Participants with highest social functioning score (<10) were more likely to achieve SVR (67%) than those with lowest social functioning (41%; OR 2.92, 95% CI 1.13–7.59, p = 0.027). In analyses adjusted for HIV status, gender, *INFL4* genotype, HCV RNA level, duration of infection, and current OST [5], social functioning score remained an independent predictor of SVR (AOR 5.11, 95% CI 1.30–20.15, p = 0.020) (Table 2).

### Longitudinal HRQoL and social functioning

**Fig 2:**
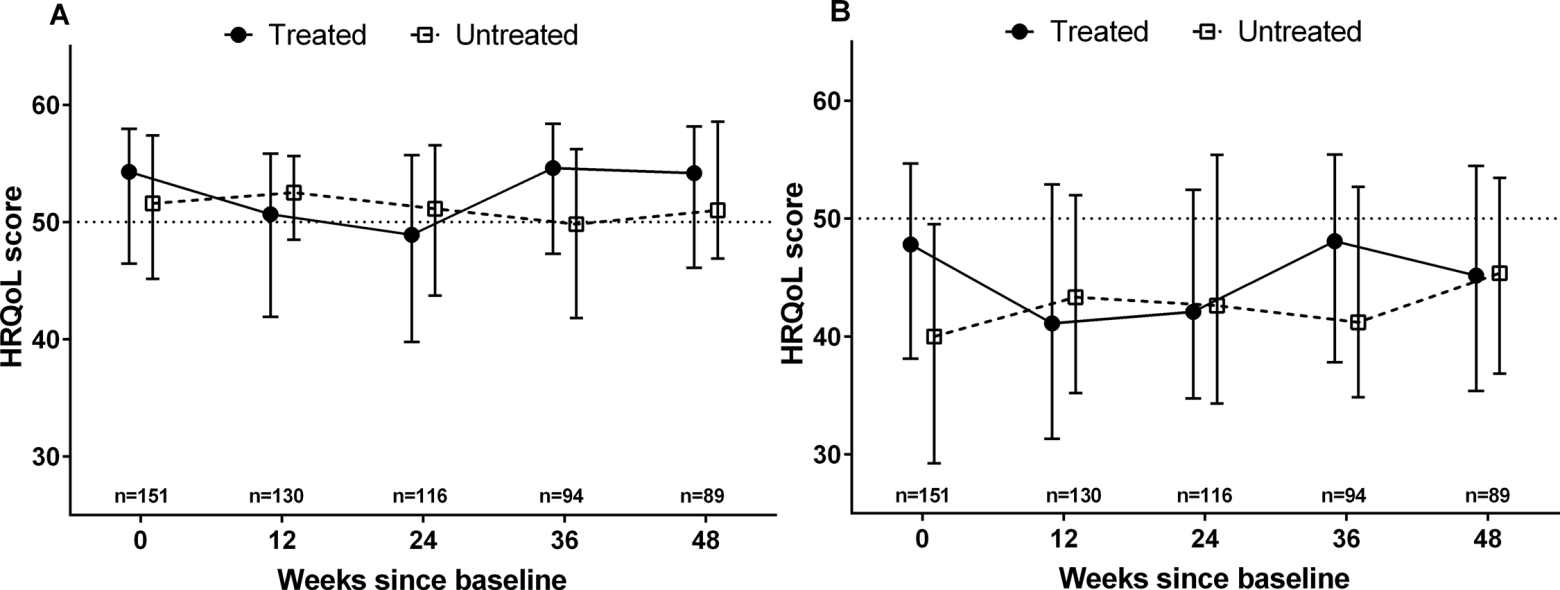
**Changes in physical (A) and mental (B) health-related quality of life after recent HCV infection, by treatment status.** Median, IQR displayed.

Physical and mental HRQoL varied during treatment from baseline to on-treatment, while no change was observed among untreated participants (Fig 2). There was a significant fall in physical HRQoL from baseline to on-treatment (54.3 vs 50.7, p<0.001), which returned to baseline levels after treatment up to week 48 (54.3 vs 54.6, p = 0.968). Mental HRQoL similarly declined from baseline to on-treatment (47.8 vs 41.1, p = 0.014) and returned to baseline after treatment (47.8 vs 46.9, p = 0.449). Crude social functioning score did not vary significantly during the study in either treated or untreated groups (S2 Fig). Baseline to post-treatment median social functioning scores were comparable (11 vs 10, p = 0.256)

In unadjusted analyses, education, employment, higher social functioning, and alcohol consumption were associated with higher physical HRQoL, and depression reduced HRQoL longitudinally (Table 3). After adjustment, higher HRQoL score was predicted by tertiary education (3.98 units, 95% CI 1.92–5.87, p<0.001), regular employment (2.99 units, 95% CI 1.48–4.50, p<0.001), alcohol >2 drinks/day (3.73 units, 95% CI 1.83–5.64, p<0.001) and lower HRQoL by depression (-2.84 units, 95% CI -4.20 to -1.14; p<0.001).

**Table 3:** Factors associated with longitudinal health-related quality of life (n = 151).

Characteristic	Physical HRQoL				Mental HRQoL			
	Univariable model		Multivariable model		Univariable model		Multivariable model	
	coeff. (95% CI)	p value	$\hat{\text{a}}$ coeff. (95% CI)	p value	coeff. (95% CI)	p value	$\hat{\text{a}}$ coeff. (95% CI)	p value
**Age** >34 (vs ≤34 yrs)	-1.25 (-3.64, 1.15)	0.307	–	–	-0.62 (-3.43, 2.21)	0.671	–	–
**Female gender** (vs male)	0.70 (-1.83, 3.22)	0.590	–	–	-1.11 (-4.09, 1.87)	0.465	–	–
**Caucasian ethnicity** (vs other)	1.38 (-2.77, 5.54)	0.514	–	–	4.89 (0.14, 9.64)	0.044	3.41 (0.78, 6.05)	0.011
**Tertiary education or greater** (vs lesser)	2.79 (0.44, 5.14)	0.020	3.98 (1.92, 5.87)	<0.001	0.95 (-1.88, 3.78)	0.510	–	–
**Regular employment** (vs irregular/none)	2.69 (1.04, 4.33)	0.001	2.99 (1.48, 4.50)	<0.001	2.33 (0.93, 4.57)	0.041	–	–
**Accommodation stable** (vs unstable)	1.40 (-1.09, 3.88)	0.272	–	–	2.32 (-1.22, 5.88)	0.198	–	–
**Social functioning, higher vs lower**^1^	2.80 (0.77, 4.83)	0.007	–	–	8.42 (6.02, 10.8)	<0.001	7.17 (5.52, 8.82)	<0.001
**OST, current** (vs none)	0.98 (-1.21, 3.17)	0.382	–	–	0.76 (-2.26, 3.78)	0.621	–	–
**Injecting drug use**, ever (vs never)	-0.62 (-3.43, 2.19)	0.665	–	–	-6.72 (-9.89, -3.54)	<0.001	-4.81 (-6.72, -2.91)	<0.001
** Injecting drug use**, in last 6mo (vs none)	0.37 (-2.36, 3.10)	0.791	–	–	-5.89 (-8.96, -2.81)	<0.001	–	–
** Injecting drug use**, in last 1mo (vs none)	-0.07 (-1.77, 1.62)	0.933	–	–	-4.13 (-6.39, -1.87)	<0.001	–	–
** Sharing needles/equipment (**vs none)	0.55 (-1.76, 2.85)	0.643	–	–	-1.10 (-4.57, 2.37)	0.534	–	–
**Alcohol use in last month** (vs none)	2.01 (0.43, 3.59)	0.013	–	–	2.23 (0.02, 4.46)	0.048	–	–
**Alcohol >2 drinks/day, last month** (vs ≤2)	3.16 (1.10, 5.22)	0.003	3.73 (1.83, 5.64)	<0.001	0.69 (-2.32, 3.69)	0.654	–	–
**Major depression current** (vs none)	-2.52 (-4.04, -1.00)	0.001	-2.84 (-4.20, -1.49)	<0.001	n/a^2^	n/a^2^	–	–
**HIV co-infected** (vs uninfected)	-0.44 (-3.03, 2.15)	0.742	- 1.78 (-3.84, 0.28)	0.090	2.76 (-0.23, 5.75)	0.071	-1.16 (-2.91, 0.59)	0.195
**Symptomatic acute HCV** (vs asymptomatic)	-1.61 (-4.39, 1.17)	0.256	–	–	0.29 (-2.88, 3.45)	0.859	–	
**Treatment** (vs no treatment)	0.17 (-2.45, 2.79)	0.901	-1.38 (-3.41, 0.64)	0.181	1.56 (-1.53, 4.66)	0.322	1.61 (-0.08, 3.31)	0.062
**Among treated:**								
** On-treatment** (vs at baseline) (n = 111)	-4.13 (-5.30, -2.96)	<0.001	-4.90 (-6.33, -3.48)	<0.001^3^	-3.64 (-4.88, -2.40)	<0.001	-3.71 (-5.55, -1.86)	<0.001^3^
** Post-treatment** (vs at baseline) (n = 111)	0.08 (-1.09, 1.25)	0.895	–	–	-1.25 (-2.48, -0.02)	0.047	0.06 (-1.16, 2.44)	0.485^3^
**HCV clearance (SVR or spontaneous)** (vs persisting viraemia)	-0.28 (-2.73, 2.18)	0.824	–	–	-1.20 (-4.11, 1.71)	0.419	–	–
***IFNL4* genotype CC** (vs non-CC)	1.21 (-1.08, 3.51)	0.300	–	–	2.35 (-0.52, 5.21)	0.109	–	–

^1^OTI score <10 vs ≥15)

^2^Omitted as covariate due to co-linearity.

^3^Treatment (vs no treatment) used in final model. Sensitivity analysis substituted on-treatment/post-treatment (vs baseline), adjusting for same covariates.

OST, opiate substitution therapy; $\hat{\text{a}}$ coeff, adjusted co-efficient.

Mental HRQoL was higher among Caucasians, regularly employed, higher social functioning, and those recently consuming alcohol, and was lower among people injecting drugs (ever, currently or actively) and on-treatment in unadjusted analyses. After adjustment, higher mental HRQoL was independently associated with Caucasian ethnicity (3.41 units, 95% CI 0.78–6.05; p = 0.011), and higher social functioning (<10 vs ≥15: 7.17 units, 95% CI 5.52–8.82, p<0.001). Lower mental HRQoL was independently associated with ever injecting drugs (-4.81 units, 95% CI -6.72– -2.91, p<0.001). Treatment commencement and HIV status did not independently predict change in either physical or mental HRQoL. However, where treatment was categorised by before-, on- and after-treatment time points, physical and mental HRQoL were both independently lower (-4.90 units, p<0.001; and -3.71 units, p<0.001, respectively) at on-treatment time points compared to baseline (Table 3).

Higher social functioning was independently associated with higher physical HRQoL (1.08 units, 95% CI 0.47–1.69, p = 0.001), mental HRQoL (1.24 units, 95% CI 0.65–1.82, p<0.001), HIV co-infection (3.31, 95% CI 2.22–4.40, p<0.001) and SVR (1.29, 95% CI 0.16–2.41, p = 0.025) (Table 4). Social functioning was lower among participants who had ever injected drugs (2.25 units, 1.08–3.41, p<0.001) or had major depression (1.36 units, 0.59–2.41, p = 0.001). Treatment and SVR were co-linear so treatment was excluded from the final model. Without adjustment for SVR, treatment was also associated with improved social functioning.

**Table 4:** Factors associated with longitudinal social functioning score (*lower* score corresponds to *higher* functioning), (n = 146).

Characteristic	Univariable model		Multivariable model	
	coeff. (95% CI)	p value	$\hat{\text{a}}$ coeff. (95% CI)	p value
**Age** >34 (vs ≤34 yrs)	-1.27 (-2.94, 0.41)	0.137	–	–
**Female gender** (vs male)	2.59 (0.83, 4.35)	0.004	–	–
**Caucasian ethnicity** (vs other)	-2.42 (-5.34, 0.50)	0.104	–	–
**Tertiary education or greater** (vs lesser)	-1.28 (-2.97, 0.41)	0.137	–	–
**Physical HRQoL >50**^**th**^ (vs ≤50^th^ centile)	-1.16 (-2.08, -0.24)	0.013	-1.08 (-1.69, -0.47)	0.001
**Mental HRQoL >50**^**th**^ (vs ≤50^th^ centile)	-2.08 (-2.98, -1.19)	<0.001	-1.24 (-1.82, -0.65)	<0.001
**Opiate substitution therapy, current** (vs none)	0.39 (-1.22, 1.99)	0.637	–	–
**Injecting drug use**, ever (vs never)	5.56 (3.78, 7.34)	<0.001	2.25 (1.08, 3.41)	<0.001
** Injecting drug use**, in last 6mo (vs none)	6.59 (4.89, 8.30)	<0.001	–	–
** Injecting drug use**, in last 1mo (vs none)	3.50 (2.39, 4.61)	<0.001	–	–
** Sharing needles/equipment**, in last 1mo (vs none)	1.40 (-0.27, 3.07)	0.100	–	–
**Alcohol use in last month** (vs none)	-1.36 (-2.46, -0.26)	0.016	–	–
**Alcohol >2 drinks/day, last month** (vs ≤2)	0.95 (-0.46, 2.36)	0.187	–	–
**Major depression current** (vs none)	3.17 (2.10, 4.23)	<0.001	1.36 (0.59, 2.14)	0.001
**HIV co-infected** (vs uninfected)	-4.79 (-6.40, -3.13)	<0.001	-3.31 (-4.40, -2.22)	<0.001
**Symptomatic acute HCV** (vs asymptomatic)	-1.85 (-3.76, 0.05)	0.057	–	–
**Treatment** (vs no treatment)	-2.69 (-4.50, -0.89)	0.003	n/a^1^	n/a^1^
**SVR** (vs no SVR)	-2.30 (-4.38, -0.28)	0.026	-1.29 (-2.41, -0.16)	0.025
**Spontaneous clearance** (vs persisting viraemia)	1.85 (-0.73, 3.76)	0.185	–	–
***IFNL4* genotype CC** (vs non-CC)	-1.34 (-3.09, 0.40)	0.130	–	–

^1^Treatment and SVR co-linear; treatment removed from final model.

$\hat{\text{a}}$ coeff, adjusted co-efficient.

## Discussion

This study makes several novel findings regarding quality of life and social functioning in the setting of recent HCV infection within a predominantly PWID cohort. First, treatment uptake and SVR are higher among those with better social functioning suggesting that supports to improve social marginalisation when embarking on HCV treatment may be beneficial. Optimising social stability may play an important role in managing acute HCV irrespective of the introduction of DAA therapies. Second, quality of life is independently influenced by 24 weeks of PEG-IFN therapy, yet these effects only persist whilst on therapy, and there is no detectable difference in HRQoL by treatment or HCV RNA status by 24 weeks post-treatment. While the decline in HRQoL is modest, PWID are a vulnerable population who would be better served by early access to IFN-free therapies. Targeted treatment of PWID should be a priority given the risk of HCV transmission through high rates of needle/syringe sharing. Third, several factors best characterised as markers of social marginalisation influence either physical or mental HRQoL: ethnicity, education, employment, depression, injecting drug use and social functioning score itself. Finally, in this Australian cohort, HIV coinfection has no adverse impact on HRQoL and is associated with higher social stability. To our knowledge, this is the only prospective data of HRQoL during recent HCV infection. Beyond application to clinical decision-making, it is of broader public health importance since it supports the need for simpler, more tolerable treatments for use in vulnerable populations particularly where tolerability is a key consideration.

Chronic HCV has been associated with lower quality of life, but the mechanism is unclear [12–18], although advanced fibrosis in chronic HCV probably drives some HRQoL decline [14–17]. Successful treatment and clearance of HCV has also been related to improvements in the physical and psychological components of HRQoL scores [14, 21, 22]. On-treatment changes in HRQoL noted in this study are in keeping with known HCV treatment side-effects [12, 20, 21, 37]. An independent association between symptomatic seroconversion and baseline physical HRQoL was also observed, which is biologically plausible given components of the HRQoL scores measure vitality, pain, and physical activity. Despite an on-treatment decline in HRQoL, the effects were short lived.

Injecting drug use was observed to independently reduce mental HRQoL, however it was not independently associated with treatment uptake, adherence or SVR in other ATAHC analyses [5, 34, 35]. Moreover, other cohort studies have documented lower HRQoL and well-being among PWID, regardless of HCV status [38, 39], and the association of injecting and HRQoL during treatment is consistent with that data.

This study observed variations in social functioning among those with HIV and PWID. The HIV subset with acute HCV was notably different to the HCV mono-infected cohort in ATAHC [36]. HIV coinfected participants were men-who-have-sex-with-men (MSM), who were more likely to acquire HCV sexually, were less marginalised, and had fewer mental health or substance use comorbidities than the HCV monoinfected participants. It is probable that these differences are related to the demographic population (MSM versus PWID) rather than HIV status. The observation of a negative relationship between PWID and social functioning and mental HRQoL may in part be due to unmeasured confounding, such as neuropsychiatric comorbidities other than depression [25].

The findings of an independent relationship between social functioning and treatment uptake and SVR probably reflect clinical and patient decisions in this non-randomised treatment study. But they suggest that with attention to appropriate support services, treatment outcomes could be improved. The OTI social functioning scale measures stable housing, changes in employment and unemployment, inter-personal conflict, and support provided by peers [23]. Clinicians and health services could address social instability in the lead up to treatment, for instance, by linking individuals with social workers for housing and employment case management, support groups for peer networks, and counselling for conflict management [40]. Assessing social function prior to treatment could prompt assessment for these social interventions on an individualised basis.

This study has a number of limitations. First, measuring HRQoL and social functioning is based on self-report and there is potential for social desirability bias particularly among those who received treatment. Nevertheless, the SF-12v2 and OTI questionnaires are validated instruments used in similar populations of with HCV infection, injecting drug use and OST services [12, 23, 33]. Second, participants were likely to be aware of their HCV RNA status through their clinical visits so the difference between virus, treatment and awareness of diagnosis effects cannot be untangled. Third, the nature of PEG-IFN-based treatment means that in a non-randomised trial, clinicians and patients do not make blinded decisions to participate. Selection biases among those receiving treatment may underestimate the negative impact of PEG-IFN on HRQoL or social stability since they were a more stable group at the outset. Fourth, recruitment and management through tertiary centres suggests this cohort may be engaged with health services, and be more stable than other populations with recent HCV. Finally, these findings may not be generalizable to other acute HCV contexts or non-injecting cohorts.

In conclusion, this study finds that social stability influences treatment uptake and virological outcome, and may continue to do so even as improved antivirals become available for recent HCV infection. The findings have implications for support services that accompany HCV treatment programs. Optimising social support might improve the proportion commencing treatment, virological response and quality of life. A comprehensive individualised assessment of social supports and factors related to social marginalisation may improve treatment outcomes. Recent HCV treatment with PEG-IFN also has a short term adverse impact on HRQoL. While this finding supports previous recommendations that injecting drug use alone should not be considered as reason to defer HCV treatment, IFN-free therapy would be ideally suited to this population. Access to tolerable therapy should become a key priority so that high-risk PWID populations can be targeted for HCV treatment to also prevent transmission.

## Supporting Information

S1 Fig Recent HCV treatment uptake, adherence, and sustained virological response. By physical (A) or mental (B) health-related quality of life (HRQoL) score (high ≥50 vs low <50) at baseline.
(TIF)

S2 Fig Changes in social functioning (Opiate Treatment Index score) after recent HCV infection, by treatment status. *Lower* score indicated *higher* social function status. Median, IQR displayed.
(TIF)

S1 Table Factors associated with higher health-related quality of life at baseline. (DOCX)

S2 Table Factors associated with higher social functioning status at baseline. (DOCX)

## Abbreviations

ATAHC: Australian Trial in Acute Hepatitis C
HCV: Hepatitis C virus
HIV: human immunodeficiency virus
HRQoL: health-related quality of life
SVR: sustained virological response
PEG-IFN: pegylated-interferon
RBV: ribavirin
PWID: people who inject drugs
OTI: Opiate Treatment Index
LLOQ: lower limit of quantification
LLOD: lower limit of detection
IQR: interquartile range
MSM: men-who-have-sex-with-men
N/A: not applicable
OR: odds ratio
aOR: adjusted odds ratio
