# Supplementary figures and images for: Quality of Life and Social Functioning during Treatment of Recent Hepatitis C Infection: A Multi-Centre Prospective Cohort
Source: PLoS One. 2016 Jun 29;11(6):e0150655. doi: 10.1371/journal.pone.0150655 (PMC4927167; doi:10.1371/journal.pone.0150655)

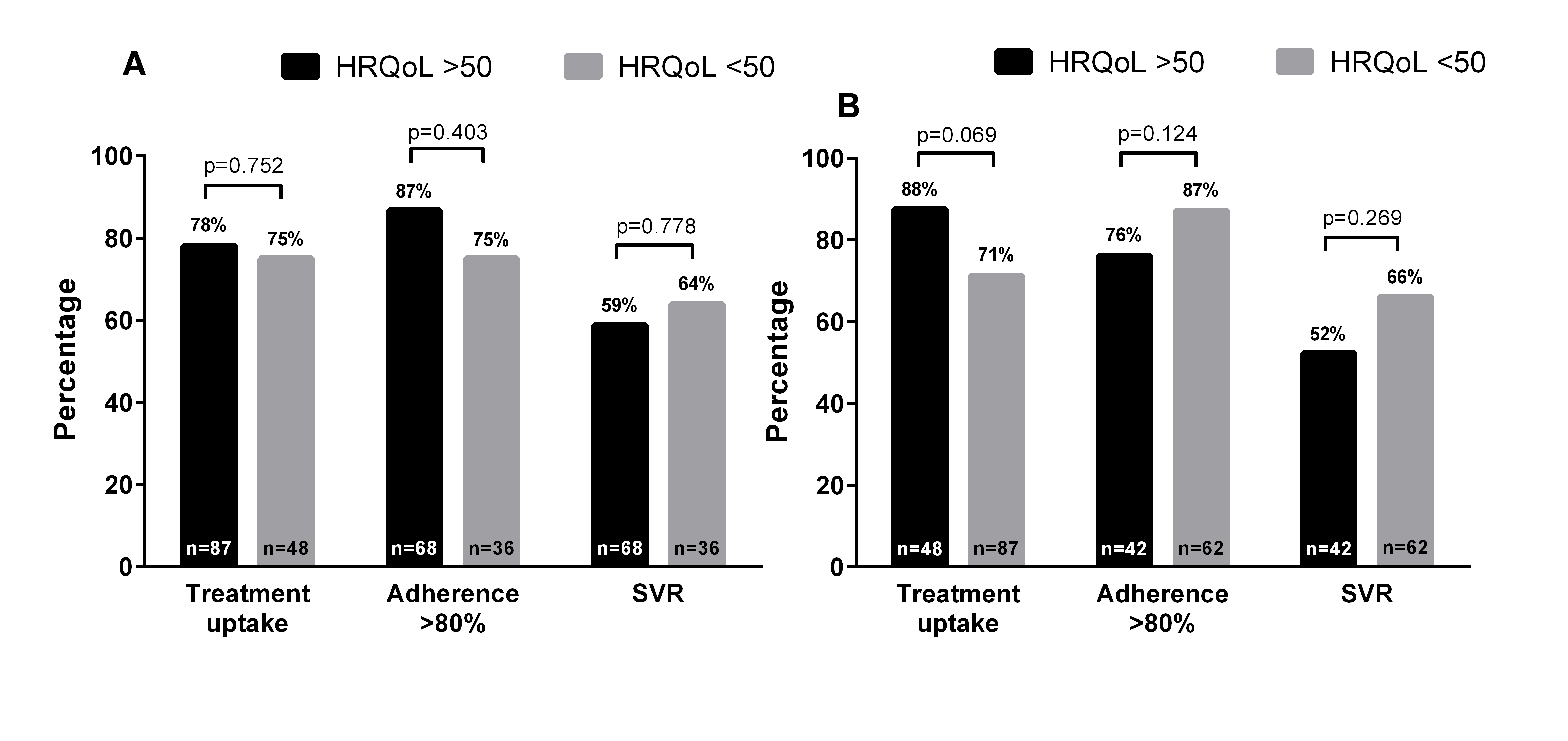

Supplement: S1 Fig — By physical (A) or mental (B) health-related quality of life (HRQoL) score (high ≥50 vs low <50) at baseline. (TIF) [file pone.0150655.s001.tif]

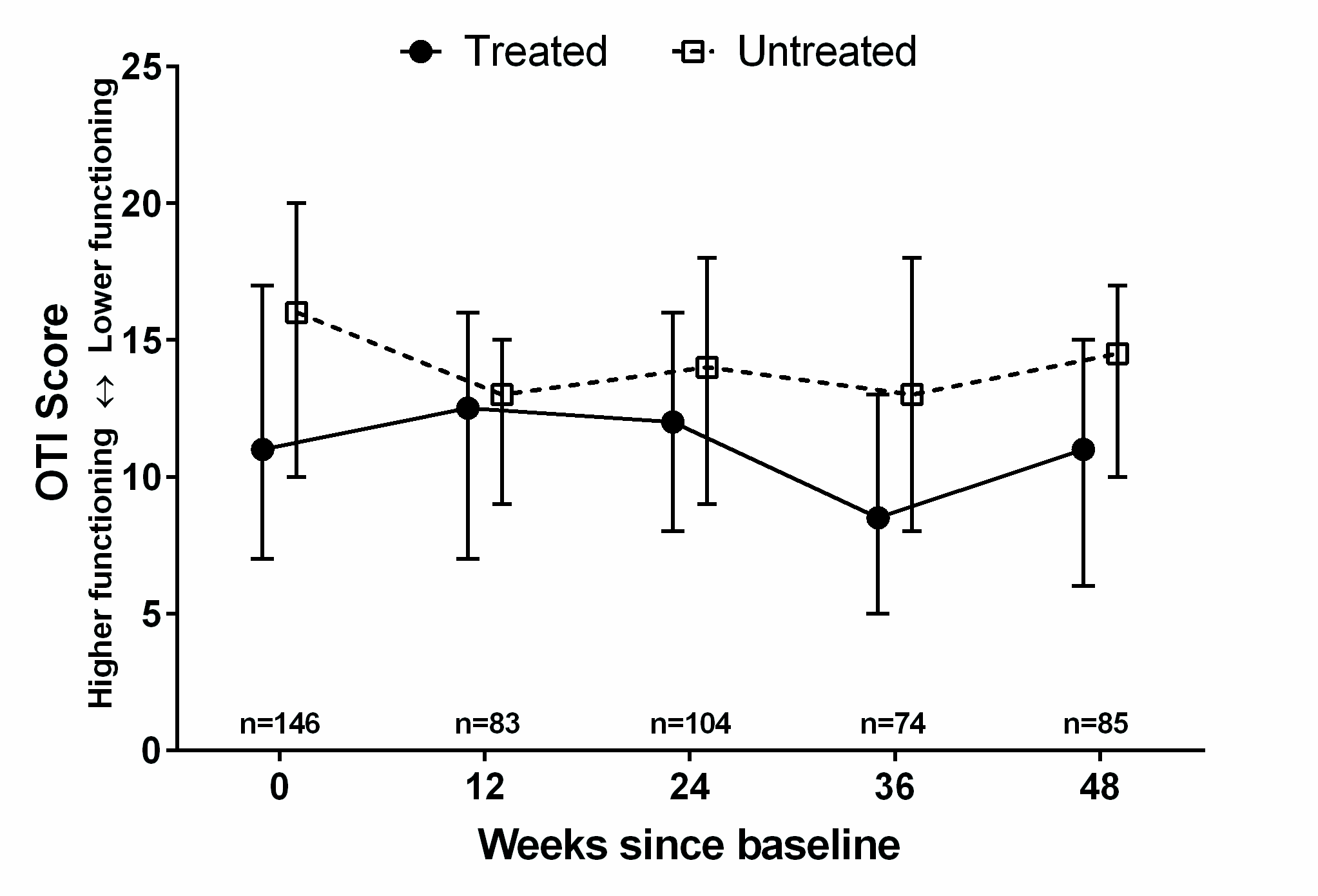

Supplement: S2 Fig — Lower score indicated higher social function status. Median, IQR displayed. (TIF) [file pone.0150655.s002.tif]
