# Supplementary material for: Quality of Life and Social Functioning during Treatment of Recent Hepatitis C Infection: A Multi-Centre Prospective Cohort
Source: PLoS One. 2016 Jun 29;11(6):e0150655. doi: 10.1371/journal.pone.0150655 (PMC4927167; doi:10.1371/journal.pone.0150655)
Supplement: S1 Table — (DOCX) [file pone.0150655.s003.docx]

**S1 Table: Factors associated with higher health-related quality of life at baseline** (>50^th^ versus ≤50^th^ centile, n=151)

| **Characteristic** | **Physical HRQoL** | | | | |  | **Mental HRQoL** | | | | |
| --- | --- | --- | --- | --- | --- | --- | --- | --- | --- | --- | --- |
|  | **Higher HRQoL**  **n (%)** | **Univariable model** | | **Multivariable model** | |  | **Higher HRQoL n (%)** | **Univariable model** | | **Multivariable model** | |
|  |  | OR (95% CI) | p value | AOR (95% CI) | p value |  |  | OR (95% CI) | p value | AOR (95% CI) | p value |
| **Age** >34 (vs ≤34 yrs) | 49 (67%) | 1.42 (0.73-2.76) | 0.301 | – | – |  | 28 (38%) | 1.32 (0.68-2.58) | 0.418 | – | – |
| **Female gender** (vs male) | 30 (65%) | 1.25 (0.62-2.52) | 0.527 | – | – |  | 18 (39%) | 1.16 (0.58-2.31) | 0.682 | – | – |
| **Caucasian ethnicity** (vs other) | 88 (64%) | 1.90 (0.60-5.42) | 0.299 | – | – |  | 48 (35%) | 0.97 (0.31-3.06) | 0.960 | – | – |
| **Tertiary education or greater** (vs lesser) | 46 (74%) | 2.35 (1.16-4.75) | 0.018 | – | – |  | 22 (35%) | 1.03 (0.52-2.03) | 0.934 | – | – |
| **Regular employment** (vs irregular/none) | 47 (80%) | 3.66 (1.72-7.81) | 0.001 | 4.51 (1.90-10.7) | <0.001 |  | 26 (44%) | 20.2 (1.01-4.03) | 0.047 | – | – |
| **Accommodation stable** (vs unstable) | 83 (63%) | 1.30 (0.42-3.96) | 0.648 | – | – |  | 46 (35%) | 1.35 (0.40-4.55) | 0.625 | – | – |
| **Social functioning, higher <10**  (vs lower ≥15) | 37 (76%) | 2.55 (1.09-5.95) | 0.030 | – | – |  | 22 (45%) | 2.78 (1.18-6.55) | 0.019 | – | – |
| **OST, current** (vs none) | 13 (65%) | 1.11 (0.41-2.97) | 0.836 | – | – |  | 9 (45%) | 1.62 (0.62-4.19) | 0.322 | – | – |
| **HCV transmission,** **injecting**  (vs sexual/other) | 66 (59%) | 0.43 (0.16-1.15) | 0.094 | – | – |  | 34 (30%) | 0.44 (0.18-1.04) | 0.061 | – | – |
| **Injecting drug use**, ever (vs never) | 95 (63%) | 0.81 (0.36-1.83) | 0.614 | – | – |  | 35 (30%) | 0.35 (0.16-0.77) | 0.010 | 0.30 (0.13-0.71) | 0.006 |
| **Injecting drug use**, in last 6mo (vs none) | 60 (63%) | 1.07 (0.52-2.21) | 0.851 | – | – |  | 29 (30%) | 0.47 (0.23-0.97) | 0.042 | – | – |
| **Injecting drug use**, in last 1mo (vs none) | 31 (58%) | 0.75 (0.38-1.50) | 0.414 | – | – |  | 13 (25%) | 0.47 (0.22-0.99) | 0.046 | – | – |
| **Sharing needles/equipment**, (vs none) | 15 (56%) | 0.83 (0.28-2.51) | 0.746 | – | – |  | 7 (26%) | 1.40 (0.38-5.16) | 0.613 | – | – |
| **Alcohol use in last month** (vs none) | 63 (68%) | 2.17 (1.06-4.45) | 0.034 | – | – |  | 30 (33%) | 0.74 (0.36-1.52) | 0.412 | – | – |
| **Alcohol**  **>2 drinks/day, last month** (vs ≤2) | 13 (72%) | 1.62 (0.54-4.83) | 0.390 | – | – |  | 6 (33%) | 0.96 (0.34-2.75) | 0.945 | – | – |
| **Major depression current** (vs none) | 13 (52%) | 0.58 (0.24-1.38) | 0.219 | – | – |  | na^1^ | – | – | – | – |
| **HIV co-infected** (vs uninfected) | 30 (70%) | 1.53 (0.72-3.25) | 0.273 | 0.96 (0.39-2.35) | 0.928 |  | 16 (37%) | 1.14 (0.55-2.37) | 0.732 | 0.76 (0.34-1.73) | 0.515 |
| **HCV RNA negative, baseline** (vs positive) | 14 (52%) | 0.57 (0.25-1.33) | 0.192 | 0.78 (0.30-2.00) | 0.606 |  | 6 (22%) | 0.47 (0.18-1.24) | 0.128 | 0.40 (0.14-1.12) | 0.081 |
| **Duration of infection >6 months at baseline** (vs ≤6 mo) | 70 (65%) | 1.33 (0.64-2.73) | 0.444 | – | – |  | 40 (37%) | 1.36 (0.64-2.90) | 0.430 | – | – |
| **Symptomatic acute HCV**  (vs asymptomatic) | 29 (48%) | 0.33 (0.15-0.71) | 0.004 | 0.25 (0.11-0.57) | <0.001 |  | 27 (44%) | 2.01 (0.94-4.28) | 0.070 | – | – |
| **ALT at presentation >100 IU/L** (vs ≤100) | 57 (63%) | 1.05 (0.53-2.05) | 0.897 | – | – |  | 32 (36%) | 1.05 (0.53-2.08) | 0.887 | – | – |
| ***IFNL4* genotype CC** (vs non-CC) | 46 (61%) | 0.81 (0.41-1.61) | 0.548 | – | – |  | 29 (39%) | 1.41 (0.71-2.82) | 0.330 | – | – |

^1^Omitted as covariate due to co-linearity

OR, odds ratio; AOR, adjusted odds ratio; OST, opiate substation therapy; ALT, alanine transaminase
