# Supplementary material for: Quality of Life and Social Functioning during Treatment of Recent Hepatitis C Infection: A Multi-Centre Prospective Cohort
Source: PLoS One. 2016 Jun 29;11(6):e0150655. doi: 10.1371/journal.pone.0150655 (PMC4927167; doi:10.1371/journal.pone.0150655)
Supplement: S2 Table — (DOCX) [file pone.0150655.s004.docx]

**S2 Table. Factors associated with higher social functioning status at baseline** (OTI score ≤12 versus >12 units^1^; n=146)

| **Characteristic** | **Higher Social Functioning**  **n (%)** | **Univariable model** | | **Multivariable model** | |
| --- | --- | --- | --- | --- | --- |
|  |  | OR (95% CI) | p value | AOR (95% CI) | p value |
| **Age**, >34 (vs ≤34 yrs) | 37 (49%) | 1.19 (0.62-2.27) | 0.606 | – | – |
| **Female gender** (vs male) | 13 (31%) | 0.36 (0.17-0.76) | 0.008 | – | – |
| **Caucasian ethnicity** (vs other) | 64 (48%) | 1.28 (0.38-4.24) | 0.686 | – | – |
| **Tertiary education or greater** (vs lesser) | 31 (52%) | 1.42 (0.74-2.77) | 0.293 | – | – |
| **Physical HRQoL >50^th^** (vs ≤50^th^ centile) | 48 (54%) | 2.20 (1.07-4.53) | 0.032 | – | – |
| **Mental HRQoL >50^th^** (vs ≤50^th^ centile) | 28 (60%) | 2.15 (1.05-4.41) | 0.036 | – | – |
| **Opiate substitution therapy, current** (vs none) | 6 (35%) | 0.57 (0.20-1.64) | 0.298 | – | – |
| **HCV transmission,** **injecting** (vs sexual) | 38 (36%) | 0.16 (0.05-0.39) | <0.001 | – | – |
| **Injecting drug use**, ever (vs never) | 43 (38%) | 0.14 (0.05-0.37) | <0.001 | 0.18 (0.06-0.59) | 0.004 |
| **Injecting drug use**, in last 6mo (vs none) | 32 (34%) | 0.22 (0.10-0.47) | <0.001 | – | – |
| **Injecting drug use**, in last 1mo (vs none) | 13 (26%) | 0.24 (0.12-0.52) | <0.001 | – | – |
| **Sharing needles/equipment**, in last 1mo (vs none) | 3 (12%) | 0.18 (0.43-0.78) | 0.022 | 0.16 (0.04-0.74) | 0.019 |
| **Alcohol use in last month** (vs none) | 48 (53%) | 2.21 (1.07-4.60) | 0.033 | – | – |
| **Alcohol**  **>2 drinks/day, last month** (vs ≤2) | 8 (47%) | 1.02 (0.37-2.82) | 0.972 | – | – |
| **Major depression current** (vs none) | 4 (17%) | 0.19 (0.06-0.59) | 0.004 | – | – |
| **HIV co-infected** (vs uninfected) | 32 (70%) | 3.89 (1.84-8.22) | <0.001 | 2.40 (1.02-5.66) | 0.046 |
| **HCV RNA positive at baseline** (vs negative) | 7 (28%) | 0.37 (0.14-0.95) | 0.039 | 0.58 (0.16-2.20) | 0.426 |
| **Duration of infection >6 months** (vs ≤6 mo) | 47 (47%) | 0.91 (0.45-1.84) | 0.792 | – | – |
| **Symptomatic acute HCV** (vs asymptomatic) | 30 (49%) | 1.43 (0.69-2.97) | 0.336 | – | – |
| **ALT at presentation >100 IU/L** (vs ≤100) | 48 (55%) | 2.11 (1.07-4.17) | 0.031 | – | – |
| ***IFNL4* genotype CC** (vs non-CC) | 33 (45%) | 0.88 (0.45-1.73) | 0.712 | – | – |

^1^Binary variable based on distribution median

OR, odds ratio; AOR, adjusted odds ratio; ALT, alanine transaminase
